# Aneurism of the Abdominal Aorta—Ligature of the Right Common Iliac

**Published:** 1866-10

**Authors:** A. J. Baxter

**Affiliations:** Chicago, Illinois


*Aneurism of the Abdominal Aorta—Ligature of the Right Common Iliac.* By A. J. Baxter, M. D., Chicago, Illinois.

Very few of the operations of surgery have been attended with more uniformly fatal results than operations upon the large arteries, and evidence becomes peculiarly valuable when it can be applied to cases, determining whether or not an operation may be advisable.

Case. Early in July last the patient first came under observation. He was a native of England, aged 37 years, of medium height, and quite muscular. His health had been uniformly good, until within three months. The patient stated that since that time a tumor had been growing in his abdomen, which materially interfered with his occupation as a laborer. It was found on examination to be a pulsating tumor, situated in the course of the abdominal aorta, and in the median line of the body, at least five inches in diameter. Auscultation developed the peculiar whirring sound, with the double character given it by the systolic and diastolic impulse of the heart. Palpation gave the true aneurismal thrill, and showed it to be elastic and bounding under pressure, with the pulsation plainly perceptible over the whole surface, and also that it was immovable from its position. When uniformly compressed, it could evidently be partially emptied of its contents, but immediately returned to its usual size when the pressure was removed. Pressure on the aorta above the tumor greatly moderated the force of its pulsation; pressure on the distal side had no such effect.

From these and other symptoms of varied importance, and after repeated examinations had left not the smallest room for doubt, it was decided to be aneurism of the abdominal aorta.

It was subsequently ascertained that he had been treated by compression over the tumor, but with no good effect. The only symptom not directly connected with the tumor itself was, that the circulation in the left lower limb was greatly reduced in force, from which was inferred that the left common iliac was either partially or wholly occluded and involved in the tumor itself, which subsequently proved to be true.

Before deciding upon an operation, the following considerations presented themselves:

1st. That the aneurism would prove fatal if left without operation.

2d. That compression had been tried without avail.

3d. That an operation afforded some chance of escaping a fatal result.

The danger accompanying the operation was fully explained, the patient fully comprehending and accepting the alternative of the operation being performed.

In considering what operation was adapted to this particular case, of course the Hunterian was wholly out of the question. The operation with the ligature on the distal side of the tumor has been practiced for more than seventy years, and has been followed by good results. That modification of it called "Wardrop's Operation" was performed, which consists of ligating one of the main branches of the diseased artery, but in reality it was what is called "Brasdor's Operation," as one of the common iliacs being closed, the ligation of the other would be essentially "Brasdor's Operation," although considered anatomically it would be Wardrop's.

The only preparation deemed necessary for the patient was a few days' rest with light diet, and to have his bowels well evacuated the day before the operation. It was decided to perform it on Sunday, 22d July. The following medical gentlemen assisted during the operation: Prof. Andrews, Chicago Medical College; Drs. McClellan, Macdonald, W. C. Lyman, Edwards, S. J. Jones and Manheimer, of this city, and Dr. W. B. Slayter, Surgeon of the City Hospital, Halifax, N. S.; also, a Committee of the St. George's Society, to which the patient belonged.

Chloroform being administered to full anæsthesia, the usual incision for this operation was made through the skin, and the abdominal muscles were successively divided down to the transversalis fascia, which was carefully cut through, and the peritoneum and ureter pushed aside, disclosing the tumor. There was little difficulty in separating the artery from the vein and nerves, and passing the ligature from without inwards. The subsequent steps of the operation are too unimportant to detail here. After the patient had recovered from his anæsthesia, he was placed in bed and the limb enveloped in cotton, to preserve the temperature. A moderate dose of morphine was administered.

On visiting the patient later in the day, pulsation in the tumor was sensibly diminished, and the temperature of the limb had not sensibly changed. Some light broth was given to the patient, and an opiate for the night, and he was left in the care of his friends. Pulsation continued to diminish in the tumor till at the end of thirty-eight hours it was wholly imperceptible; at the same time, circulation in the right limb having been interrupted by the ligature, was being rapidly restored through the various anastomoses, pulsation and a normal temperature being re-established.

At the end of the second day there was slight tympanites, which passed away in another day, leaving everything favorable to a good result, as the various functions of the body were normally performed.

Everything being thus favorable, a good result was anticipated, but such was not to be the case.

On Thursday, four days after the operation, some morphia was prescribed in powders of one-half grain each, one to be given at night, and repeated if necessary. Through the culpable negligence, disobedience and ignorance of the person in charge of the patient for the time, they were given repeatedly, in the absence of the physician, till fatal narcotism was produced. Every effort made by the physicians who were summoned proved unavailing, and he died from narcotic poisoning, and not as a consequence of the operation.

